# Vitamin D, serum 25(OH)D, LL-37 and polymorphisms in a Canadian First Nation population with endemic tuberculosis

**DOI:** 10.3402/ijch.v74.28952

**Published:** 2015-08-19

**Authors:** Linda Larcombe, Neeloffer Mookherjee, Joyce Slater, Caroline Slivinski, Joe Dantouze, Matthew Singer, Chris Whaley, Lizette Denechezhe, Sara Matyas, Kate Decter, Emily Turner-Brannen, Clare Ramsey, Peter Nickerson, Pamela Orr

**Affiliations:** 1 Department of Internal Medicine, University of Manitoba, Winnipeg, MB, Canada; 2 Department of Medical Microbiology, University of Manitoba, Winnipeg, MB, Canada; 3 Department of Community Health Sciences, University of Manitoba, Winnipeg, MB, Canada; 4 Manitoba Centre for Proteomics and Systems Biology, University of Manitoba, Winnipeg, MB, Canada; 5 Department of Human Nutritional Sciences, University of Manitoba, Winnipeg, MB, Canada; 6 Northlands Denesuline First Nation, Lac Brochet, MB, Canada

**Keywords:** vitamin D, indigenous, genetics, cathelicidin (LL-37), tuberculosis

## Abstract

**Background:**

Canadian First Nation populations have experienced endemic and epidemic tuberculosis (TB) for decades. Vitamin D–mediated induction of the host defence peptide LL-37 is known to enhance control of pathogens such as *Mycobacterium tuberculosis*.

**Objective:**

Evaluate associations between serum levels of 25-hydroxy vitamin D (25(OH)D) and LL-37, in adult Dene First Nation participants (N = 34) and assess correlations with single nucleotide polymorphisms (SNPs) in the vitamin D receptor (VDR) and vitamin D binding protein (VDBP).

**Design:**

Venous blood was collected from all participants at baseline (winter and summer) and in conjunction with taking vitamin D supplements (1,000 IU/day) (winter and summer). Samples were analysed using ELISA for concentrations of vitamin D and LL-37, and SNPs in the VDR and VDBP regions were genotyped.

**Results:**

Circulating levels of 25(OH)D were not altered by vitamin D supplementation, but LL-37 levels were significantly decreased. VDBP and VDR SNPs did not correlate with serum concentrations of 25(OH)D, but LL-37 levels significantly decreased in individuals with VDBP D432E T/G and T/T, and with VDR SNP Bsm1 T/T genotypes.

**Conclusions:**

Our findings suggest that vitamin D supplementation may not be beneficial as an intervention to boost innate immune resistance to *M. tuberculosis* in the Dene population.

Canadian Aboriginal (indigenous) populations, which include First Nations, Metis and Inuit peoples, suffer from a high burden of morbidity and mortality from both infectious (including tuberculosis (TB), respiratory syncytial virus and influenza) and chronic (including diabetes mellitus and autoimmune) diseases (1–4). Increasing attention to the role of nutrition in the health and disease in Canadian Aboriginal people has led to the recognition of the prevalence of overt hunger as well as the “hidden hunger” of micronutrient deficiency (5–7). Vitamin D insufficiency is common in industrialized and developing nations, and low circulating levels of vitamin D have been associated with a higher risk of infections including active TB (8–10). Low serum vitamin D levels have been reported in First Nation populations as are clinical manifestations of hypovitaminosis D including rickets in children and elevated fracture risk with low mineral bone density in women (5–7, 11, 12). Risk factors for hypovitaminosis D include northern latitude, food insecurity and poverty. Vitamin D (from food, sunshine and supplements), in particular, has a wide spectrum of activity affecting calcium and bone metabolism, immune and cardiovascular systems, regulation of cell proliferation and glucose metabolism (13).

Like many northern Canadian indigenous communities, the Northlands Denesuline (Dene) First Nation have experienced endemic TB for many years (1, 14). Previous studies have demonstrated that the Canadian Dene populations have low winter levels of serum 25-hydroxy vitamin D (25(OH)D) and high vitamin D binding protein (VDBP) (5). We previously demonstrated that vitamin D supplementation resulted in enhanced innate immune responses. In particular, cytokines (interleukin-6, -12 and -23) required to control intracellular pathogens such as *Mycobacterium tuberculosis* (Mtb) were upregulated in macrophages isolated from Canadian Caucasians in response to a lipoprotein antigen TLR2/1L but not in macrophages from the Dene (15). TLR2/1L is a synthetic lipoprotein of *M. tuberculosis*, the causative agent of TB. TLR2/1L has been shown to induce the expression of vitamin D receptor (VDR) and the enzyme CYP27B1 required for the synthesis of the active metabolite 1,25(OH)_2_D of vitamin D (16).

It is currently unclear to what extent, if any, vitamin D levels and metabolism may play in susceptibility/resistance to TB among Dene peoples. Vitamin D metabolites induce the expression of the human antimicrobial host defence peptide, cathelicidin LL-37. The promoter region of the *hCAP18* gene, which encodes for LL-37, contains a vitamin D response element (17, 18). Induction of LL-37 expression has been shown to be mediated by interaction of VDR and its ligand 1,25(OH)_2_D, the active form of vitamin D (17). LL-37 is a naturally occurring host defence peptide with both antimicrobial and immunomodulatory properties required for the control of infections, including TB (19, 20). Inadequate levels of 25(OH)D suppress the expression of *hCAP18*/LL-37 and consequently may compromise immune responses to infections(8, 9, 10, 20). Consistent with this, low plasma levels of LL-37 have been associated with increased susceptibility to infections (21). It is, therefore, possible that impaired immune response to infections may also be due to dysregulation of the vitamin D metabolism pathway and consequently inadequate induction of the peptide LL-37.

Single nucleotide polymorphisms (SNPs) in the genes for VDBP and VDR are associated with altered vitamin D metabolism and impaired immune response to infections (22, 23). SNPs at restriction enzyme sites D432E and T436K are known to change the VDBP protein structure resulting in the 3 most common variants of VDBP, Gc1f, Gc1s and Gc2. These variants differentially influence the bioavailability and binding affinity of VDBP to 25(OH)D, thereby affecting vitamin D-mediated induction of the LL-37 (22–25). Similarly, 4 VDR SNPs at restriction enzyme sites, Fok1, Bsm1, Apa1, and Taqα1, have been associated with vitamin D-related disease conditions and susceptibility to infectious diseases (10). Studies linking SNPs of VDBP and VDR to altered immune responses to infections among First Nation populations are limited.

In this prospective study, we examined specific VDBP and VDR SNPs and their correlation with circulating serum levels of vitamin 25(OH)D and LL-37, before and after vitamin D supplementation (1,000 IU/day), in a Dene cohort. Correlations between serum levels of 25(OH)D and LL-37 with VDBP and VDR SNPs may provide insight into factors associated with increased prevalence of TB in the Dene.

## Materials and methods

### Ethics statement

First Nation research principles of ownership, control, access and possession (OCAP) were followed, and the study had the support of the community's Chief and Council (26). Study participants were 18 years of age or older and provided informed written consent as required by the University of Manitoba Health Research Ethics Board.

### Study participants

This study is part of a long-term research partnership with the community of Northlands Denesuline First Nation (Dene) located at Lac Brochet, 58° latitude in northern Manitoba, Canada. Recruited participants self-identified as Dene, an Aboriginal group that is part of the larger Athapaskan-speaking language family, which has a northern branch including the Gwich'in and Tlingit and a southern branch including the Apache and Navajo. The community comprised 145 census families and 605 individuals (255 of whom are >19 years of age) (27).

The study was advertised widely in the community and the Research nurse screened volunteers. Community consultation determined that convenience sampling (volunteers responding to advertisement), rather than random sampling, was the only methodology acceptable for this research. Inclusion criteria included the following: age ≥18 years, informed consent, Dene self-identification, commitment to participate for the duration of the study and commitment to refrain from self-administering vitamin D supplements >400 IU/day. Individuals self-administering a low dose of vitamin D (≤400 IU/day) were allowed to participate in the study. Volunteers were excluded from the study if they had a current or recent infection or any autoimmune conditions at the time of screening. Those taking medication that affects vitamin D catabolism (i.e. anti-TB medications, prednisone, seizure drugs) were also excluded from the study. Each participant agreed to complete a food frequency questionnaire (FFQ) and provide blood samples at winter and summer time points over a 2-year period.

### Baseline phase (pre-vitamin D supplementation)

In the first year of the study (baseline), data collected in winter (January–March) and late summer (September–October) included age, sex, body mass index (BMI), medications, chronic health conditions and self-identified ethnicity. A modified FFQ was administered at 2 time points to assess the available vitamin D containing market foods (i.e. milk, margarine, etc.) and traditional foods (i.e. local fish, caribou fat, meat and organs). The frequency of vitamin D containing foods and portion sizes were assessed using the vitamin D values from the Canadian Nutrient File [CNF, (28)]. For wild foods for which there were no vitamin D values in the CNF, the value from a reasonable comparison food was used (i.e. beef was used for caribou tongue, heart, liver, kidney and fat) (6). Self-administered supplementary vitamin D intake was added into the calculation of dietary intake. The questionnaire was field-tested and administered at winter and summer time points. Participants were asked to recall food consumption patterns for the previous month.

### Vitamin D supplementation phase

In the second year of the study, tablets containing 1,000 IU of vitamin D_3_ were provided to all participants in blister packs to be taken daily for a 12-month period beginning in the winter. The participants were reminded to take the tablets using phone calls, post cards, in-person visits from the study team and local radio announcements. The FFQ was also administered during the winter and summer post-vitamin D supplementation. The blister packs of used and unused vitamin D tablets were collected to measure adherence. Percent compliance was calculated based on the total number of vitamin D daily doses taken over the total length of the study period.

### Analyses of serum 25-hydroxyvitamin D and LL-37 concentrations

Venous blood samples were collected into SST tubes (BD Vacutainer Systems) within a 2-week period at the winter and summer visits during year 1 and 2 of the study. Samples were centrifuged within 2 h at 1,200×*g* for 15 min, and the serum was separated and stored at −20°C. Serum concentrations of 25(OH)D (AC-57F1; 25-hydroxy vitamin D, EIA Immunodiagnostic Systems, Inc., Scottsdale, AZ, USA) and LL-37 (HK321; Human LL-37 ELISA Kit, Hycult Biotechnology, Uden, the Netherlands) were evaluated by ELISA as per the manufacturer's instructions. Serum samples were diluted 1:40 for 25-OHD ELISA, and 2 technical replicates were assayed for each diluted serum sample. For the 25-OHD_3_ ELISA, intra- and inter-plate coefficients of variation were 2.2±2.9% and 5.7±1.4%, respectively. Serum concentrations of 25(OH)D that were ≥75 nmol/L were considered minimally required to contribute to immune responses to infections (29).

### SNP determination

Genomic DNA was extracted from buffy coats of blood samples using the QIAamp DNA Blood Mini Kit (Qiagen, Inc., Toronto, ON, Canada). SNPs at VDBP restriction sites D432E (rs7041) and T436K (rs4588), and VDR restriction sites Fok1 (rs10735810), Bsm1 (rs1544410), Apa1 (rs7975232), and Taqα1 (rs731236) were analysed as follows: PCR conditions for VDBP amplification in a 25 µl reaction: 3.5 µl of extracted DNA; 2.5 µl of 10× PCR buffer; 1.0 µl of 50 mM MgSO_4_; 0.2 µl of 10 mM dNTP mix; 16.2 µl of reagent grade water; 0.1 µl of Platinum^®^ Taq (Invitrogen Life Technologies Corp., Burlington, ON, Canada); and 0.5 µl of each of the forward primer and reverse primers. PCR conditions were 95°C for 15 min followed by 35 cycles of 94°C for 20 s, 58°C for 20 s, and 72°C for 20 s with a 1s increment for each subsequent cycle, and 1 cycle at 72°C for 10 min. PCR amplification of VDR SNPs Bsm1 (T/C), Apa1 (G/T), Taqα1 (C/T) and Fok1 (T/C) was performed using published protocols and primers (30). Analysis of the VDR SNPs at the restriction sites Bsm1 (B/b (T/C)), Apa1 (A/a (T/G)), Taqα1 (T/t (T/C)), Fok1 (F/f (C/T)) and VDBP SNPs at D432E (G/T) and T436K (C/A) were detected using RFLP and were visualized with ethidium bromide staining and ultraviolet.

### Statistical analyses

Data management and statistical analyses were performed using GraphPad Prism V.6.0 (GraphPad Software, Inc., La Jolla, CA, USA). Data were evaluated for normality using D'Agostino and Pearson omnibus normality test. Outliers were identified and removed using robust regression and outlier removal (Q=1%). Comparison of mean concentrations of 25(OH)D and LL-37 was evaluated using the paired 2-tailed Student's t-test. Linear regression was used to evaluate the relationship between vitamin D supplementation and serum concentrations of LL-37 and 25(OH)D. The potential relationship between serum concentrations of 25(OH)D and LL-37 was evaluated in the context of compliance by taking vitamin D supplements in year 2. A p-value of 0.05 or less was considered statistically significant.

## Results

### Study participants

**Fig. 1:**
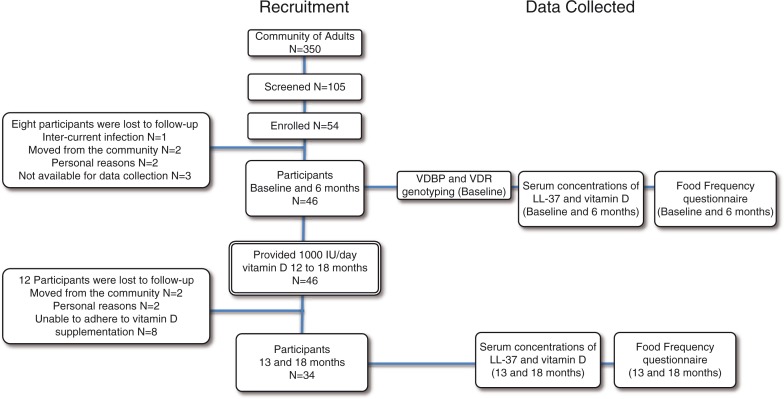
Flow of the 18-month study, participant recruitment and retention, and data collection. The study began in January, therefore, baseline (month 0), and month 13 data collection points were in the winter. The data collection points at months 6 and 18 were in the summer.

In a community of 330 adults, 105 volunteers were screened for inclusion (Fig. 1). Fifty-one were excluded because they were either taking medications that affect vitamin D absorption, had current or recent infections during the course of the study, or taking >400 IU/day vitamin D. Fifty-four participants were enrolled in the study in year 1, of which 8 participants were lost to follow-up in year 1. In year 2 of the study, 12 participants were lost to follow-up due to either moving to another community, and/or inability to adhere to vitamin D supplementation. A total of thirty-four participants, 16 males (42 years ±15, BMI 29.6±6) and 18 females (43 years ±11, BMI 29.7±5.8), completed the two-year study. All data were analysed taking compliance into consideration. The mean percentage compliance for 1,000 IU/day vitamin D supplementation among all 34 study participants over the entire year was 54% (0.54±0.39). There were no significant differences in any of the data analysed between the total number of participants (n = 34) and those who were either ≥80% (n=14) or 100% (n=6) compliant. Therefore, data presented in this study are from participants who completed the 2-year study (n=34).

### Analyses of vitamin D intake

**Fig. 2:**
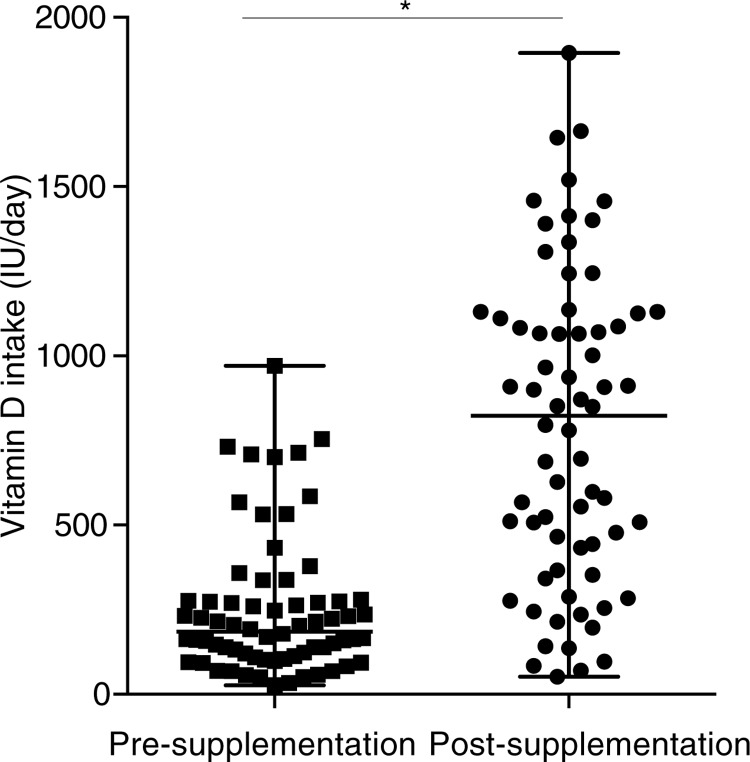
Vitamin D intake significantly increased with vitamin D supplementation. Vitamin D intake was measured using a modified food frequency questionnaire (FFQ). Median and range of vitamin D intake (IU/day) are shown for pre- and post-vitamin D supplementation (*p < 0.05). An FFQ was administered at 4 time points (at baseline, 4, 13 and 18 months) to assess the available vitamin D containing market foods (i.e. milk, margarine, etc.) and traditional foods (i.e. local fish, caribou fat, meat and organs). The frequency of vitamin D containing foods and portion sizes were assessed using the vitamin D values from the Canadian Nutrient File 2010 (28).

The FFQ analyses showed that vitamin D intake was increased significantly on post vitamin D supplementation (Fig. 2). Our previous analyses had demonstrated that vitamin D intake from food sources did not change pre- and post-vitamin D supplementation (6). This suggests that the mean increase in vitamin D intake in this study was largely due to vitamin D supplementation during year 2 of the study.

### Vitamin D supplementation did not alter circulating levels of 25(OH)D

**Fig. 3:**
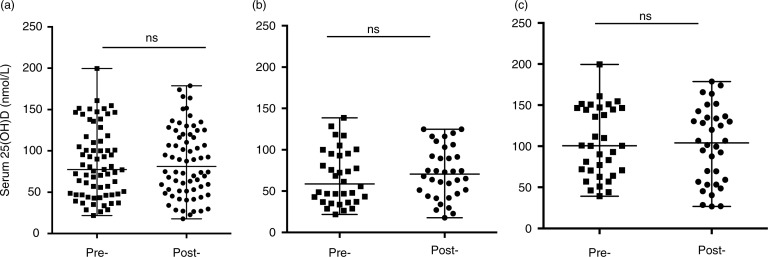
Vitamin D supplementation did not alter circulating levels of 25(OH)D. Serum concentration of 25(OH)D was monitored by ELISA. Median and range of serum 25(OH)D are shown for pre- and post- vitamin D supplementation (a) without seasonal breakdown, or with seasonal analyses in either (b) winter or (c) summer (ns = non-significant).

Circulating levels of 25(OH)D were monitored in serum for all the participants (n=34), before and after vitamin D supplementation. Vitamin D supplementation did not significantly change the median serum concentrations of 25(OH)D in the participants (Fig. 3a). Consistent with this, seasonal analyses also demonstrated that vitamin D supplementation did not alter the circulating levels of 25(OH)D in summer (Fig. 3b) or winter (Fig. 3c). There was no significant correlation between the vitamin D intake with circulating levels of 25(OH)D either pre- or post-supplementation (Supplementary Fig. 1a). There was also no significant correlation between serum 25(OH)D and the rate of compliance of taking vitamin D supplements (Supplementary Fig. 1c).

### Vitamin D supplementation significantly altered circulating levels of LL-37

**Fig. 4:**
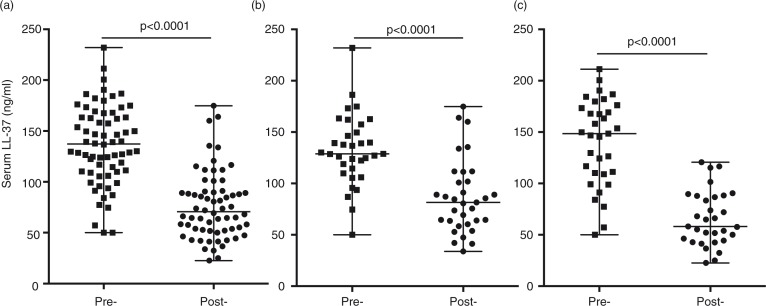
Vitamin D supplementation significantly decreased circulating serum LL-37 levels. Serum concentration of LL-37 was monitored by ELISA. Median and range of serum LL-37 are shown for pre- and post-vitamin D supplementation (a) without seasonal breakdown, or with seasonal analyses in either (b) winter or (c) summer (ns = non-significant).

Serum concentration of LL-37 was monitored by ELISA in all the participants (n = 34), pre- and post-vitamin D supplementation. Serum levels of LL-37 significantly decreased after vitamin D supplementation (Fig. 4a). Seasonal analyses also showed that the decrease in LL-37 post-vitamin D supplementation was significant both in winter and summer (Fig. 4b and 4c), indicating that seasonal bioavailability of vitamin D did not have an impact on the decrease of LL-37. However, there was no significant correlation between serum levels of LL-37 with either vitamin D intake (Supplementary Fig. 1b) or rate of compliance of vitamin D supplements (Supplementary Fig. 1d), pre- or post-vitamin D supplementation.

### Association of VDBP SNPs with circulating levels of 25(OH)D and LL-37

**Fig. 5:**
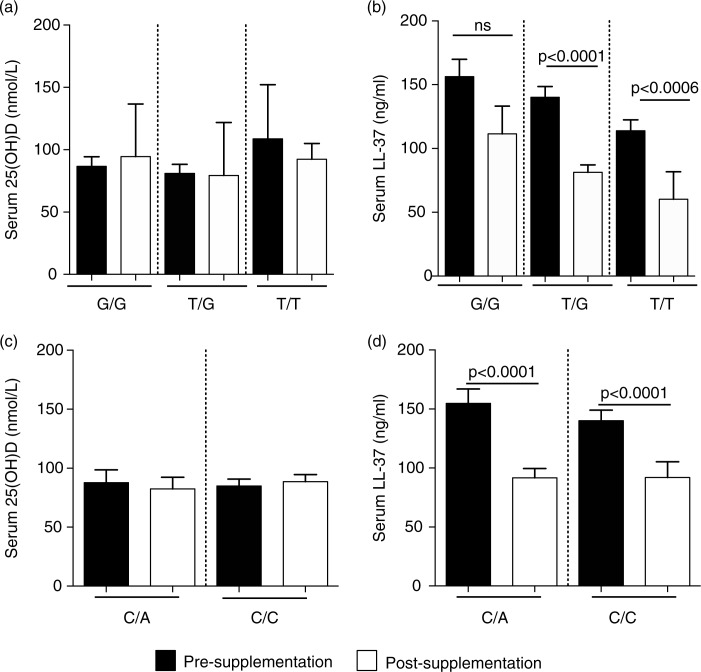
Association of VDBP SNPs with serum concentration of 25(OH)D and LL-37: specific VDBP SNPs were analysed at D432E (G/T) and T436K (C/A) restriction sites. The figure shows the association of the VDBP SNPs at D432E site with serum concentration of (a) 25(OH)D and (b) LL-37, and at T436K restriction site with serum concentration of (c) 25(OH)D and (d) LL-37, pre- and post-vitamin D supplementation.

Previous studies have indicated that specific VDBP polymorphisms, especially the *GC* genotype, affect binding affinity to 25(OH)D and may be associated with vitamin D deficiency. SNPs, in exon 11 of the VDBP gene located on the long arm of chromosome 4, are responsible for the 2 most common genetic variants – D432E (NCBI rs7041) and T436K (NCBI rs4588). These 2 gene polymorphisms produce variant proteins that differ in their affinity for vitamin D (22, 31). Consequently, this may also alter downstream effector molecules induced in response to vitamin D, such as the induction of cathelicidin LL-37. We have previously shown that the Dene have a high frequency of specific VDBP SNPs associated with dysregulation of Th1-immune responses required for resolution of infections (5). In this study, we examined the association of VDBP SNPs (D432E and T436K) with serum concentrations of 25(OH)D and LL-37, pre- and post-vitamin D supplementation. Mean serum concentrations of 25(OH)D were not significantly different when compared by VDBP restriction sites D432E (Fig. 5a) and T336K (Fig. 5c). In contrast, serum concentrations of LL-37 significantly decreased post-vitamin D supplementation in individuals with D432E T/G and T/T genotypes, but not in those with the G/G genotype (Fig. 5b). Similarly, serum concentration of LL-37 significantly decreased in individuals with T436K C/A and C/C genotypes (Fig. 5d).

### Association of VDR SNPs with circulating levels of 25(OH)D and LL-37

**Fig. 6:**
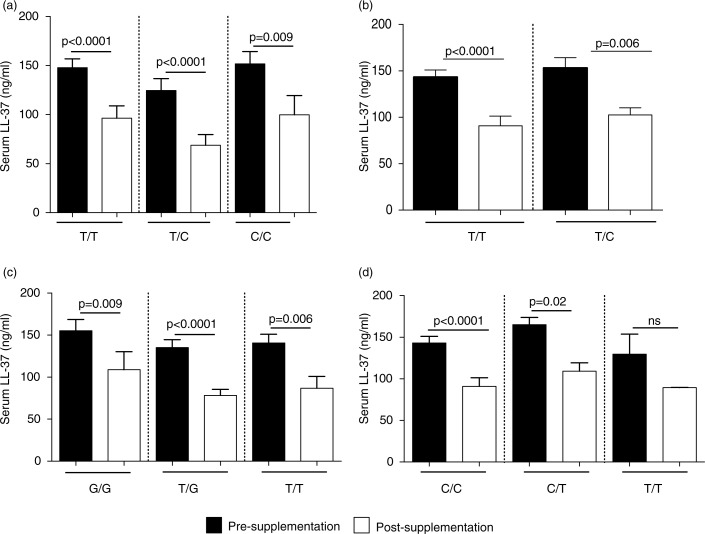
Association of VDR SNPs with serum concentration of LL-37: specific VDR SNPs were analysed at specific 4 restriction sites (a) Fok1, (b) Taqα1, (c) Apa1 and (d) Bsm1. The figure shows the association of the VDR SNPs with serum levels of LL-37 monitored by ELISA, pre- and post-vitamin D supplementation.

Previous studies have demonstrated that specific VDR SNPs are associated with vitamin D-related disease conditions and susceptibility to infectious diseases. In this study, we monitored the association of VDR SNPs at the restriction sites Bsm1 (B/b (T/C)), Apa1 (A/a (T/G)), Taqα1 (T/t (T/C)), Fok1 (F/f (C/T)), with serum concentrations of 25(OH)D and LL-37, pre- and post-vitamin D supplementation. Concentrations of 25(OH)D were not significantly different in pre- and post-vitamin D supplementation for any VDR genotype monitored (Supplementary Fig. 2). Serum levels of LL-37 significantly decreased in individuals with all VDR genotypes monitored, except for individuals with Bsm1 (T/T) genotype (Fig. 6).

Dene participants with the VDBP D432E G/G genotype had higher levels of circulating LL-37 compared to those with the “T” allele. The serum concentration of LL-37 did not decrease significantly after vitamin D supplementation for participants who were homozygous D432E-G/G. All VDR SNPs monitored in this study were associated with significant decrease in serum LL-37 concentration post-vitamin D supplementation, except for participants with the VDR Bsm1 T/T genotype. The serum concentration of LL-37 significantly decreased after vitamin D supplementation except for a subset of study participants, specifically those with the VDBP D432E G/G and VDR Bsm1 T/T genotypes. Our results suggest that the vitamin D-mediated regulation of microbial resistance may be influenced by VDBP and VDR gene variants in the Dene population.

## Discussion

This is the first prospective study in a Dene population to examine the effects of vitamin D supplementation on the circulating levels of 25(OH)D and LL-37, and its association with specific VDBP and VDR SNPs. Vitamin D supplementation for 8 months in the study participants did not significantly alter the serum levels of 25(OH)D (Fig. 1). This is in contrast to previous studies demonstrating that within 3 months of vitamin D supplementation, serum 25(OH)D is significantly higher compared to pre-supplementation baseline levels (32, 33). Vitamin D supplementation has been shown to provide a protective effect against respiratory infections (32). One of the mechanisms of vitamin D-mediated protection against respiratory infections, primarily TB, is suggested to be by enhancing the expression and the antimicrobial activity of the human cathelicidin LL-37 (18, 32). In a small healthy population, there was a positive relationship between serum 25(OH)D and LL-37 levels leading to the supposition that vitamin D supplementation would raise the systemic levels of LL-37 (34). In this study, levels of LL-37 significantly decreased following vitamin D supplementation in the Dene (Fig. 2). Furthermore, there was no correlation between circulating levels of 25(OH)D and LL-37, in contrast to previous studies demonstrating positive correlations between circulating cathelicidin and 25(OH)D (35).

Functional variability in the metabolism of vitamin D may be due to specific SNPs associated with various signalling intermediates within the vitamin D pathway. Gene polymorphisms in both VDBP and VDR has been suggested to affect the bioavailability and abundance of 25(OH)D, therefore, the optimum concentration of circulating 25(OH)D required for disease resolution may vary (22). In a previous study, we demonstrated that the Dene had a high frequency of specific VDBP and VDR SNPs associated with an ineffective Th1-immune response to *M. tuberculosis*, and may be a risk factor for susceptibility to disease (5). In this study, we further examined specific SNPs in VDBP and VDR, and their association with circulating levels of 25(OH)D and LL-37, pre- and post-vitamin D supplementation and found no significant association between serum 25(OH)D concentrations and either VDBP or VDR gene variants (Figs. 5 and 6, respectively). Whereas alteration of circulating of levels of LL-37 varied depending on specific SNPs monitored. Overall, our results suggest that vitamin D metabolism may be differently regulated in the Dene, thus affecting the induction of the downstream antimicrobial effector molecule LL-37. The mechanisms associated with the decrease of circulating cathelicidin levels following vitamin D supplementation need to be further elucidated.

Dene particiants with the VDBP D432E G/G genotype demonstrated higher levels of circulating LL-37. The serum concentration of LL-37 did not decrease significantly after vitamin D supplementation for participants who were homozygous for the D432E-G allele. All VDR SNPs monitored in this study were associated with significant decrease in serum LL-37 concentration post-vitamin D supplementation, except for participants with the VDR Bsm1 T/T genotype. As the serum concentration of LL-37 did not significantly decrease after vitamin D supplementation in only a subset of study participants, specifically those with the VDBP D432E G/G and VDR Bsm1 T/T genotypes, our results suggest that the vitamin D-mediated regulation of microbial resistance may be influenced by VDBP and VDR gene variants in the Dene population.

There may be several limitations to these study results. The sample size of this study (n = 34) even though relatively small represents 13.3% of adults in the community and 6.2% of the entire adult Dene population in Manitoba. The generalizability of results to other non-participant members of the community, or to Dene people in other communities, may be limited by the sample size and by the potential selection bias due to the recruitment method considered culturally appropriate by the community. Furthermore, the overall compliance of vitamin D supplementation in this study was around 54%. We acknowledge that this may have reduced the power of the study. Despite using specific tactics to encourage adherence to the vitamin D supplementation regimen, reasons for non-compliance included difficulties in remembering to take the tablets on a daily basis and forgetting the tablets when travelling. This is consistent with the previous studies that reflect that health concerns are often superseded by other priorities in this population, who struggle to meet basic needs (36). Participation rates in research studies are often low in First Nation communities, due to a number of factors including issues of historical mistrust, exploitation, differing perceptions and priorities (7, 35). The issues of recruitment, retention and compliance in this study reflect the reality of health intervention studies in First Nation populations.

Assessing potential associations in First Nations between low vitamin D levels and infectious diseases, such as TB, is also complicated by multiple, potentially confounding variables including housing, social (such as poverty, racism, educational and health service barriers) and biologic factors (6, 36). Nevertheless, the results of this study raise the possibility that metabolism of the vitamin D pathway and associated gene variants may be a contributing factor to the high incidence of infections such as TB in the Dene population. This study also raises doubt regarding the potential of vitamin D supplementation to boost innate immune resistance to *M. tuberculosis* in the Dene First Nation population.

## Supplementary Material

Vitamin D, serum 25(OH)D, LL-37 and polymorphisms in a Canadian First Nation population with endemic tuberculosis
