# Supplementary material for: Vitamin D, serum 25(OH)D, LL-37 and polymorphisms in a Canadian First Nation population with endemic tuberculosis
Source: Int J Circumpolar Health. 2015 Aug 19;74:10.3402/ijch.v74.28952. doi: 10.3402/ijch.v74.28952 (PMC4543449; doi:10.3402/ijch.v74.28952)

Supplemental Figure 1: Serum concentrations of **(A)** 25(OH)D and **(B)** LL-37 were evaluated for potential correlations with vitamin D intake, pre- and post-vitamin D supplementation. (C) Correlation of serum concentrations of 25(OH)D and LL-37 was determined with the rate of compliance for taking vitamin D supplements.

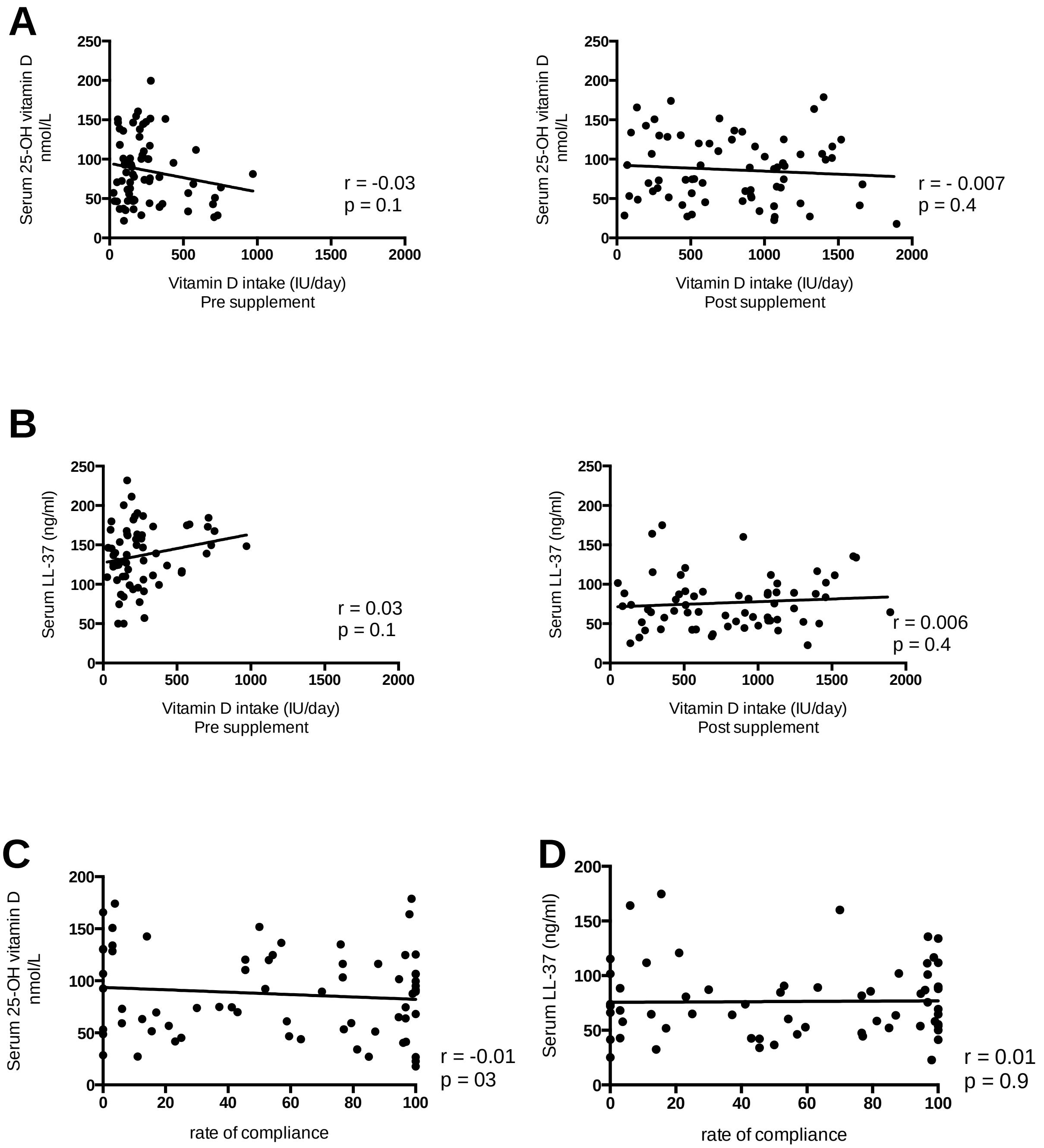

Supplemental Figure 2: Specific VDR SNPs were analyzed at specific four restriction sites **(A)** Fok1, **(B)** Taq1, **(C)** Apa1, and **(D)** Bsm1. The figure shows the association of the VDR SNPs with serum levels of 25(OH)D monitored pre- and post-vitamin D supplementation.

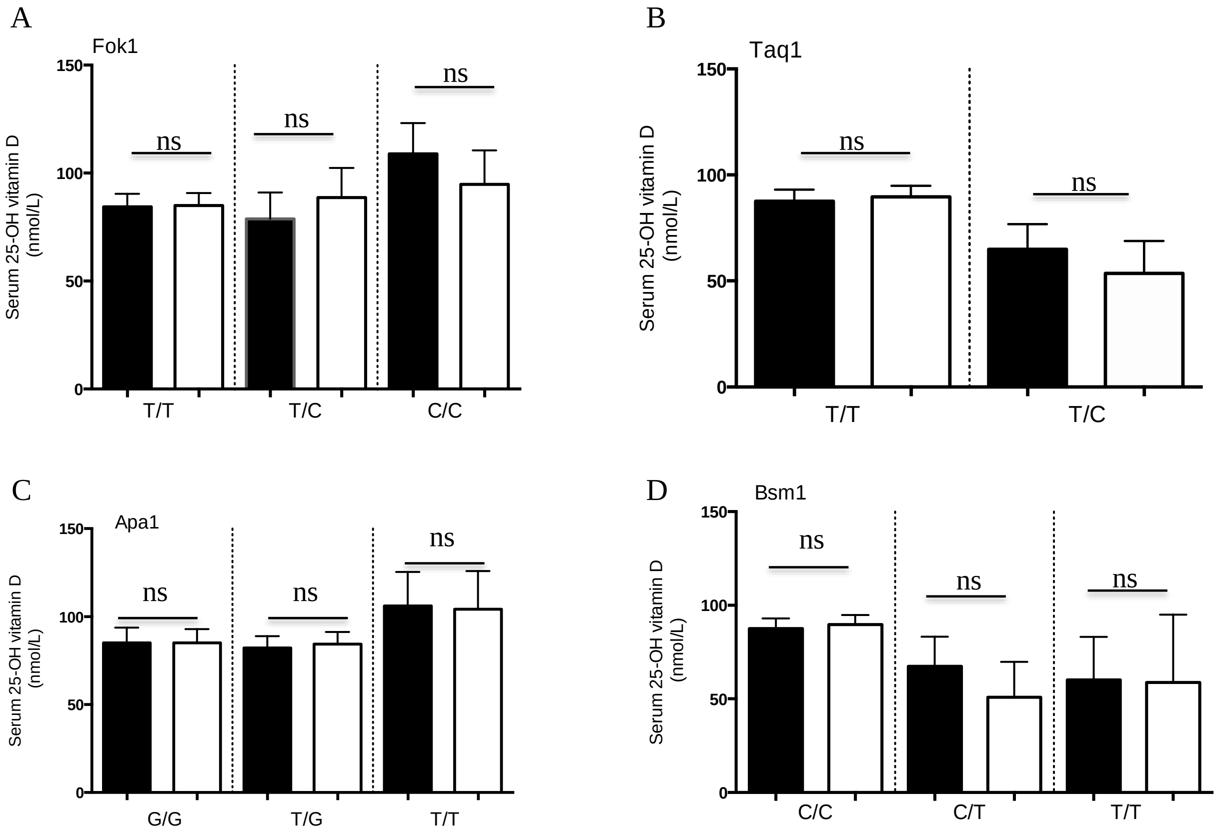

Supplement: Vitamin D, serum 25(OH)D, LL-37 and polymorphisms in a Canadian First Nation population with endemic tuberculosis [file IJCH-74-28952-s001.pdf]
